# Lactate and Myocardiac Energy Metabolism

**DOI:** 10.3389/fphys.2021.715081

**Published:** 2021-08-17

**Authors:** Shuohui Dong, Linhui Qian, Zhiqiang Cheng, Chang Chen, Kexin Wang, Sanyuan Hu, Xiang Zhang, Tongzhi Wu

**Affiliations:** ^1^Department of General Surgery, Qilu Hospital of Shandong University, Jinan, China; ^2^Department of Colorectal and Anal Surgery, Feicheng Hospital Affiliated to Shandong First Medical University, Feicheng, China; ^3^Department of General Surgery, The First Affiliated Hospital of Shandong First Medical University, Jinan, China; ^4^Adelaide Medical School and Centre of Research Excellence in Translating Nutritional Science to Good Health, The University of Adelaide, Adelaide, SA, Australia; ^5^Endocrine and Metabolic Unit, Royal Adelaide Hospital, Adelaide, SA, Australia

**Keywords:** myocardium, cardiac metabolism, energy substrate, lactate, lactate shuttle theory, myocardial ischemia, heart failure, diabetic cardiomyopathy

## Abstract

The myocardium is capable of utilizing different energy substrates, which is referred to as “metabolic flexibility.” This process assures ATP production from fatty acids, glucose, lactate, amino acids, and ketones, in the face of varying metabolic contexts. In the normal physiological state, the oxidation of fatty acids contributes to approximately 60% of energy required, and the oxidation of other substrates provides the rest. The accumulation of lactate in ischemic and hypoxic tissues has traditionally be considered as a by-product, and of little utility. However, recent evidence suggests that lactate may represent an important fuel for the myocardium during exercise or myocadiac stress. This new paradigm drives increasing interest in understanding its role in cardiac metabolism under both physiological and pathological conditions. In recent years, blood lactate has been regarded as a signal of stress in cardiac disease, linking to prognosis in patients with myocardial ischemia or heart failure. In this review, we discuss the importance of lactate as an energy source and its relevance to the progression and management of heart diseases.

## Introduction

The heart is an efficient bio-pump of high energy demand. Adenosine triphosphate (ATP) is the direct source of energy that supports the contraction and relaxation of the myocardium. ATP can be derived through the processes of oxidation and fermentation, during which the intermediate pyruvate is transformed into lactate. The latter has long been considered to be a by-product of glucose metabolism. Over the last several decades, amounting research has demonstrated that lactate is a major energy substrate for skeletal muscle, heart and brain (Gertz et al., 1988; Bergman et al., 1999b; Glenn et al., 2015), as well as a main gluconeogenesis precursor (Bergman et al., 2000; Meyer et al., 2002a,b; Emhoff et al., 2013b) and a signaling molecule (Hashimoto et al., 2007). Increased levels of blood lactate are also associated with poor outcomes in critical systemic diseases, including severe trauma, hypoxemia, septic shock and so forth (Cerovic et al., 2003; Nguyen et al., 2004; Khosravani et al., 2009). However, the biochemical and clinical significance of lactate within the field of myocadiac metabolism remains under-appreciated, reflecting an incomplete understanding of its production, transport, metabolism and biological functions (Garcia-Alvarez et al., 2014). Herein, we discuss myocardial energy metabolism, with an emphasis on the role of lactate metabolism and its relevance to the progression and management of heart diseases, including acute myocardial ischemia, and heart failure, and on diabetic state.

## Historical View on Myocardial Energy Metabolism

The initial understanding of cardiac function was first described by a Greek philosopher Aristotle. According to the latter, the heart produces necessary heat to maintain life, and cessation of the heartbeat means absence of life (Beloukas et al., 2013). This philosophy led to the recognition of energy metabolism being central to cardiac function and the capacity of cardiomyocyte to utilize various substrates to provide energy.

Oxygen was identified as a basic element of cardiac metabolism and function as early as in the 18th century (Gh, 2006). Subsequently, carbohydrate was described as an energy substrate during cardiac contraction (Locke and Rosenheim, 1907), but contributed no more than a third to the total cardiac energy demand (Evans, 1914). This observation stimulated the search of other energy substrates for myocadiac energy metabolism, which had been advanced rapidly since the introduction of coronary sinus catheterization technique (Bing et al., 1947). In this way, the oxygen extraction ratios can be computed to reflect the aerobic catabolism of different substrates. In 1954, Bing and his colleagues assessed the utilization of glucose, lactate, pyruvate, fatty acids, ketones and amino acids of the heart *in vivo* (Bing et al., 1947; Bing, 1954), and showed that fatty acids were the major energy substrate of the human heart, accounting for 67% of the total usage of oxygen. Surprisingly, lactate, formerly known as a metabolic waste product and fatigue agent, contributed 16.5% to the total usage of oxygen, in a comparable degree to glucose (17.9%) (Bing, 1954).

Subsequent studies concentrated on energy substrate usage of the heart under different circumstances. Keul et al. (1965a, b) found that, in humans during moderate intensity exercise, the contribution of fatty acids fell from 34 to 21%, while the contribution of lactate increased from 29 to 62%. In anesthetized dogs, the contribution of lactate to cardiac oxidative energy production increased to 87% when the arterial lactate concentration exceeded 4.5 mmol/L (Drake et al., 1980). These studies suggest that lactate competes with fatty acids for cardiac oxygen consumption. In recent years, the emergence of dual carbon-labeled carbohydrate isotope technique has allowed myocardial substrate utilization to be quantified precisely in humans. Based on this technique, it was observed that the myocardial isotopic lactate uptake increased from 34.9 μmol/min at rest to 120.4 μmol/min at 5 min of moderate intensity exercise in healthy male subjects (Gertz et al., 1988). This result further confirmed lactate as an important energy substrate for the heart, particularly under stress. It is now widely accepted that the heart uses various energy substrates to generate ATP, which is key to the maintenance of normal cardiac function under different circumstances.

## Myocardial Lactate Metabolism

As discussed, in addition to fatty acids, carbon sources, including glucose and lactate, are important energy substrates of the myocardium. Traditionally, it has been thought that full oxidation of glucose to CO_2_ provides most cells energy in human body, and lactate is only a product of incomplete oxidation in the face of urgent energy demands. However, if this was the case, whole-body glucose consumption would dominate over lactate consumption, and lactate production would be equivalent to its clearance (as a precursor of hepatic and renal gluconeogenesis). Rather, lactate has a circulatory turnover flux approximately twice that of glucose on a molar basis during fasting (Dunn et al., 1976; Katz et al., 1981; Stanley et al., 1986; Wolfe, 1990). Modern studies have proved that lactate could be produced continuously under aerobic conditions, and be used as an important energy source for the heart (Brooks, 2018). Accordingly, “lactate shuttle theory” was proposed to describe the transport and function of lactate within the body: lactate could act as the vehicle linking glycolysis and oxidative metabolism, and the linkages between lactate “producer” and “consumer” exist within and among cells, tissues, and organs (Brooks, 2018). In this section we will discuss the development of lactate shuttle theory in the context of myocardial energy metabolism.

### Tissue-Tissue and Cell-Cell Lactate Transport

Benefiting from the development of differential arterio-venous metabolite analysis and radiotracer techniques, the whole process of circulating lactate production and disposal was characterized in several animal studies (Brooks et al., 1984). In rats, intravenous injection of ^14^C-lactate resulted in exhaled breath gas containing ^14^CO_2_, providing early evidence for the concept of circulating lactate in contributing to energy metabolism (Brooks et al., 1973; Brooks and Gaesser, 1980). To better explore the production and disposal of circulating lactate across diverse physiological states, Donovan CM and Brooks GA subsequently recorded rates of lactate disposal in rodent models both at rest and during exercise with radiotracer techniques (Brooks and White, 1978; Brooks and Donovan, 1983; Donovan and Brooks, 1983). By measuring the quantity and activity of O2, CO2, circulating carbon sources (glucose and lactate) and other metabolites during resting and exercise (Brooks et al., 1977), the authors observed in rats that the rates of lactate flux in circulating peripheral blood were unanticipatedly high in the resting state, although to a lesser extent when compared to glucose flux. However, during exercise, the rates of lactate flux in circulating blood were observed to increase above those of glucose flux.

These phenomena are likely to have reflected the breakdown of glycogen and an increase in the production of lactate during exercise. However, the latter was not necessarily accompanied by increased blood lactate concentrations (Brooks and Donovan, 1983; Donovan and Brooks, 1983), which can be attributed to the markedly increased lactate metabolic clearance rate during exercise. Hence, relatively stable blood lactate concentration during exercise can be explained by a greater lactate metabolic clearance rate due to increased lactate oxidation and gluconeogenesis. Such adaption can avoid metabolic acidosis during exercise. As technology improves, non-invasive and non-radiating methods are available for evaluation of glucose and lactate metabolism in the human body, and the aforementioned effects of exercise on glucose and lactate flux rates have also been consistently replicated (Stanley et al., 1985; Mazzeo et al., 1986; Bergman et al., 1999a,b, 2000; Emhoff et al., 2013a,b). Fluctuations of lactate in the blood indicate that lactate transport between different tissues *via* the circulatory system. However, the main producers and consumers of blood lactate underlying these changes remain unknown.

In the following research, investigators attempted to assess tissue specificity of lactate metabolism (Katz et al., 1981; Stanley et al., 1986; Gertz et al., 1988; Bergman et al., 1999a,b). Two earlier studies in the 1970s found that lactate concentrations in red skeletal muscles in motion were lower than white skeletal muscles and their blood supplies (Mole et al., 1973; Hooker and Baldwin, 1979). Although these phenomena seemed unexplained at that time, this provided early evidence to suggest working skeletal muscles as a source of the circulating lactate. In 1988, the utilization of lactate released from working muscles by heart as a carbon source was observed (Gertz et al., 1988), which marked an important milestone for research in lactate metabolism. Subsequently, lactate transport between white and red skeletal muscles (Stanley et al., 1986; Bergman et al., 1999a), and between working skeletal muscles and heart were gradually identified (Katz et al., 1981; Gertz et al., 1988; Bergman et al., 2009). It is now well-documented that the beating heart takes up, and oxidizes lactate as a consumer in the process of the lactate transport.

The potential for lactate to transport across neighboring cells and tissues has been reported over two decades. It was initially found to occur in astrocytes-neurons in brain and fibroblasts-cancer cells in tumors (Lisanti et al., 2013; Magistretti and Allaman, 2015). Recently, mouse cardiomyocytes and fibroblasts co-culture models have shown the fibroblasts-cardiomyocytes lactate transport (Wisniewski et al., 2015; Gizak et al., 2020), supporting the concept that cardiomyocytes and fibroblasts form metabolic syncytia to share energy substrates, including lactate, and exchange molecular signals. Neighboring lactate transport improves energy metabolism efficiency and orchestrates substrate utilization in tissues. Such a metabolic architecture enables metabolic adaptability and plasticity. Nevertheless, accurate mechanisms of lactate transport between cardiomyocytes and fibroblasts are still unclear.

### Transmembrane and Intracellular Lactate Transport

The increasing recognition of tissue-tissue and cell-cell lactate transport has provided a strong impetus to understand the metabolic fate of lactate transport and utilization within the cell. The discovery of membrane lactate transporters provided a plausible explanation of the transmembrane lactate transport. Lactate oxidation rates comply with Michaelis-Menten kinetics, suggesting that cellular uptake and release of lactate are facilitated by membrane transporters. The membrane lactate transporter was first observed on rat sarcolemmal vesicles in 1990 (Roth and Brooks, 1990), and subsequently named as monocarboxylate transport (MCT) (Garcia et al., 1994).

Hitherto, various isoforms of MCT have been identified to account for intracellular lactate transport, but how is lactate utilized in the cell, and the site at which the intracellular lactate utilization takes place remains debated. Previous studies on humans and various mammals demonstrated that intracellular lactate metabolism consumes oxygen (Depocas et al., 1969; Donovan and Brooks, 1983; Stanley et al., 1985; Mazzeo et al., 1986). Lactate oxidation for energy supply especially prominent when heart or muscle is in load condition (Gertz et al., 1981, 1988; Stanley et al., 1986; Bergman et al., 1999b). With this in mind, issues arose about exactly where lactate oxidation happened within a working cardiomyocyte. Given that lactate dehydrogenase (LDH), a key enzyme to catalyze the inter-conversion of lactate and pyruvate, is widely present in the cytosol, lactate oxidation was first considered to take place in the cytosol. However, the results from some studies were inconsistent (Laughlin et al., 1993; Chatham et al., 2001). In working muscles-beating heart lactate syncytium, increased rates of lactate flux were found to accompany by augmented blood flow and oxygen consumption. In addition, when ^13^C-pyruvate was injected into myocardial blood circulation, the peaks of cytosolic ^13^C-pyruvate and ^13^C-lactate were observed. However, when ^13^C-lactate was injected directly, ^13^C-pyruvate was not detected in the cytosol (Chatham et al., 2001).

**FIGURE 1:**
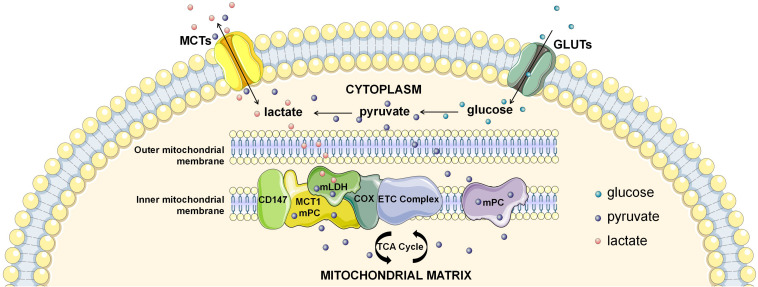
The intracellular lactate shuttle. Both extracellular uptake and glycolytic production make up the cytosolic lactate pool. Intracellular lactate is shuttled from the cytoplasm to mitochondria for subsequent oxidization, facilitated by the mitochondrial lactate oxidation complex (mLOC). Monocarboxylate transporter 1 (MCT1) is inserted into the inner mitochondrial membrane, and CD147 is an indispensable chaperone protein of MCT1. Mitochondrial LDH (mLDH) is distributed on the surface of the inner mitochondrial membrane, and oxidizes lactate to pyruvate. In addition, terminal electron transport chain element cytochrome oxidase (COX) is responsible for the endergonic lactate oxidation. Mitochondrial pyruvate carrier (mPC) is distributed in the inner mitochondrial membrane, which is responsible for the mitochondrial transmembrane transport of pyruvate.

If not in the cytosol, where does intracellular lactate oxidation occur? Another candidate location for intracellular lactate oxidation is the mitochondria, the pivotal organelle for energy metabolism. Lactate oxidation was observed in several mitochondrial preparations (Kline et al., 1986; Brandt et al., 1987; De Bari et al., 2004; Passarella et al., 2014), of which mitochondrial preparations from human skeletal muscles provided the strongest evidence (Jacobs et al., 2013). Lactate oxidative capacity of muscle mitochondria was subsequently confirmed by magnetic resonance spectroscopy imaging (MRSI) (Park et al., 2015; Chen et al., 2016). However, some studies using the same experimental settings showed inconsistent results, challenging the lactate oxidative capacity in the mitochondria (Popinigis et al., 1991; Sahlin et al., 2002). This discrepancy might be related to the separation process of mitochondria (Kirkwood et al., 1986; Glancy et al., 2017), which is highly dependent on the isolated system and susceptible to be contamination and/or disturbance. Therefore, confirmation by more reliable experiments is still required due to its conceptual importance. More recently, identification of the mitochondrial lactate oxidation complex (mLOC) with techniques of organelle purification and magnetic resonance spectroscopy imaging has provided solid evidence on mitochondrial transmembrane transport of lactate mitochondria as a key site of intracellular lactate oxidation (Figure 1; Park et al., 2015; Chen et al., 2016). Components of the mLOC include the MCT1, CD147, LDH, terminal electron transport chain element cytochrome oxidase (COX), pyruvate dehydrogenase (PDH), Krebs cycle related-enzymes, and mitochondrial respiratory chain (De Bari et al., 2004; Hashimoto et al., 2006, 2008; Atlante et al., 2007; Sonveaux et al., 2008; Passarella et al., 2014). Mitochondrial MCT1 is located in the inner membrane and is responsible for mitochondrial transmembrane transport of lactate, and CD147 is an indispensable chaperone protein of MCT1 (Park et al., 2015; Chen et al., 2016). Mitochondrial LDH was initially discovered in the sperm, but is now revealed to also exist in the liver, kidney and heart (Kline et al., 1986; Brandt et al., 1987; Tempia et al., 1988). Lactate oxidative capacity is strongly associated with the expression of mitochondrial LDH (Tempia et al., 1988), and is also associated with COX (Hashimoto et al., 2006). Finally, lactate is transformed to the end product (CO_2_) by PDH, Krebs cycle related-enzymes, and mitochondrial respiratory chain catalysis. Future research in order to better define the mLOC is eagerly anticipated.

Another issue regarding intracellular lactate metabolism is why cells generate and consume lactate at the same time. For example, cardiomyocytes release glycolytically derived lactate and take up extracellular lactate simultaneously (Goodwin et al., 1998; Bartelds et al., 1999; Emhoff et al., 2013a). Although some studies indicated that glycolytically derived pyruvate is preferentially shifted to lactate rather than to acetyl-CoA, pyruvate derived from exogenous lactate tends to form acetyl-CoA (Barnard et al., 1971; Chatham et al., 1999). To account for these observations, one plausible explanation is that pathways of glycolytic lactate production and oxidation of exogenous lactate are functionally separate in the cardiomyocyte, i.e., compartmentation of intracellular lactate metabolism (Barnard et al., 1971; Mowbray and Ottaway, 1973; Sinniah, 1978; Lewandowski, 1992). LDH reversibly catalyzed the conversion of pyruvate to lactate or lactate to pyruvate. Given that the equilibrium constant for LDH is far in the direction of lactate and the change in free energy is large, glycolytically derived pyruvate can easily shift to lactate, rather than enter the TCA cycle (Barnard et al., 1971; Rabinowitz and Enerback, 2020). In addition, myocardial mitochondrial abundance is greatest at the subsarcolemmal surface (Palmer et al., 1977). Therefore, when exogenous lactate enters cardiomyocytes, it is expected to be readily transported into the mitochondria and enters the TCA cycle. This explanation reconciles *in vivo* and *in vitro* observations relating to myocardial lactate generation and consumption (Wisneski et al., 1985; Gertz et al., 1988; Mazer et al., 1990; Goodwin et al., 1998; Bartelds et al., 1999), and is in keeping with the lactate shuttle theory (Brooks et al., 1999; Brooks, 2000). It is recently proposed a hypothesis that characteristics of intracellular lactate metabolism enables the uncoupling of mitochondrial energy generation from glycolysis, and confers cells with increased metabolic flexibility (Rabinowitz and Enerback, 2020). Future studies of intracellular lactate metabolism are needed to investigate the regulation of lactate uptake and efflux and assess the exact value of the compartmentation of lactate metabolism.

## Lactate Metabolism in Myocardial Diseases

In order to be compatible with complicated and volatile physiological and pathological states, the heart has evolved into an “omnivore” to consume different energy substrates in varying proportions. In myocardial diseases, there are universal disorders in energy substrate utilization and metabolic flexibility. While ATP production in the myocardium is often impaired in different pathological states, there is less consensus as to what actual switches in energy substrate preference occur. Traditionally, lactate has been regarded as an undesirable metabolite and has, accordingly, been used to as a biomarker of myocardial injury. However, later studies suggests that lactate is of greater relevance than other metabolic substrates, to the maintenance of metabolic flexibility in the metabolically unhealthy heart (Hutter et al., 1984; Lopaschuk et al., 2010; Garcia-Alvarez et al., 2014), raising a fundamental question: *is lactate in heart disease a savior or a devil?* This section will discuss lactate oxidation in the context of acute myocardial ischemia, heart failure and diabetes.

### Acute Myocardial Ischemia

Acute myocardial ischemia is a common feature of acute critical cardiac events including acute coronary syndrome, cardiogenic shock, and cardiac arrest. There is usually a clear etiology and the course of disease is often brief. Sudden heart attacks lead to drastic changes of cardiac metabolic environment within a short period of time. Myocardium death is mainly caused by deprivation of oxygen and energy substrates in acute myocardial ischemia (Ong et al., 2010). While fatty acids remain the main energy substrate in the ischemic myocardium (Stanley, 2001), respiration and oxidative phosphorylation functions of mitochondria are markedly impaired during ischemia. There is evidence that the number of mitochondria is augmented in acute myocardial ischemia (Ide et al., 2001), which might represent a compensatory response for acute ischemia and hypoxia. It has been proved that the most striking metabolic switch event in the ischemic myocardium relates to increased glycolysis (Trueblood et al., 2000; Askenasy, 2001; Russell et al., 2004; Carvajal et al., 2007). However, the raw materials for glycolysis in acute myocardial ischemia are in association with the extent of ischemia. During low-flow conditions, glucose uptake and lactate release may be maintained in ischemic myocardium, and increased glycolysis depends on higher influx of glucose and increased activity of glycolytic enzymes (King and Opie, 1998a,b; Horman et al., 2012; Herzig and Shaw, 2018). When blood flow is completely interrupted, glucose is replaced by glycogenolysis (Smeele et al., 2011; Wu et al., 2011). Despite profound differences in the sources of glycolytic substrates, activating or prolonging glycolysis has been shown to be beneficial for myocardial salvage in both conditions (Vanoverschelde et al., 1994; Lochner et al., 1996; Fiolet and Baartscheer, 2000; Trueblood et al., 2000; Askenasy, 2001; Russell et al., 2004; Carvajal et al., 2007; Kim et al., 2011; Smeele et al., 2011; Wu et al., 2011; Timmermans et al., 2014; Vanoverschelde et al., 1994; Lochner et al., 1996; King and Opie, 1998a,b; Fiolet and Baartscheer, 2000; Trueblood et al., 2000; Askenasy, 2001; Russell et al., 2004; Carvajal et al., 2007; Kim et al., 2011; Smeele et al., 2011; Wu et al., 2011; Horman et al., 2012; Timmermans et al., 2014; Herzig and Shaw, 2018).

The accumulation of lactate in the ischemic myocardium provides an important source of energy; both uptake and use of lactate by the myocardium increase significantly in the acute ischemic heart (Hutter et al., 1984; Lopaschuk et al., 2010). In animal shock models, lactate deprivation is related to increased mortality, while exogenous lactate infusion is associated with myocardial salvage (Barbee et al., 2000; Revelly et al., 2005; Levy et al., 2007).

Several observational studies have demonstrated that hyperlactatemia is associated with a poor prognosis in patients with acute coronary syndrome (Lazzeri et al., 2010; Vermeulen et al., 2010; Kossaify et al., 2013). In patients who received percutaneous coronary intervention, plasma lactate measured after percutaneous coronary intervention is a reliable predictor for mortality (Lazzeri et al., 2009; Valente et al., 2012). In cardiogenic shock, the prognostic value of lactate has been controversial. Some studies identified increased lactate as an independently prognostic factor (Weil and Afifi, 1970; Chiolero et al., 2000; Koreny et al., 2002; Attana et al., 2013b), however, others did not (Geppert et al., 2006). Moreover, elevated lactate levels are positively associated with mortality in cardiac arrest (Donnino et al., 2007; Nolan et al., 2008; Cocchi et al., 2011; Andersen et al., 2013). Accordingly, a large body of research is in support of lactate as a prognostic factor in acute myocardial ischemia. It is regrettable that there is yet no consensus about the cut-off values for lactate that would be associated with worse outcome. In some recent observational studies, serial measurements of lactate have been shown to be more efficient than a single measurement for outcome prediction in acute myocardial ischemia (Attana et al., 2013a).

Based upon the evidence to date, it appears that lactate has the potential to act both as an “energy substrate” and a “prognostic factor.” Numerous studies have shown benefits in preserving the function of ischemic myocardium by modifying cardiac energy substrates and increasing ATP production (Dyck et al., 2004, 2006; Ussher and Lopaschuk, 2008; Lionetti et al., 2011). Both promoting the utilization of glucose and reducing the β-oxidation of fatty acids have been proposed as anti-ischemic strategies (Stanley et al., 2005; Dyck et al., 2006). However, there is a lack of clinical evidence to support treatment with lactate supplementation or deprivation in patients with acute cardiac events. It should also be noted that observational studies do not allow to conclude on the causal relationship between lactate and clinical outcomes.

### Heart Failure

Heart failure is a complex disease which represents the end-stage outcome for many cardiac and systemic diseases (Savarese and Lund, 2017). Despite the heterogeneity of etiology (Jessup and Brozena, 2003), heart failure is associated with a marked reduction in the production of energy (Hearse, 1979; Neubauer et al., 1997; Neubauer, 2007). Impaired mitochondrial structure and oxidative function have been reported in the failing heart (Casademont and Miro, 2002; Neubauer, 2007; Aubert et al., 2013; Fukushima et al., 2015). Along with these changes, alterations of energy substrates were also detected (Krahe et al., 1993; Collins-Nakai et al., 1994; Lopaschuk et al., 2010). For example, decreasing ratio of fatty acids oxidation was observed in pressure overload-induced rat failing heart as well as mouse gene-knockout failing heart models (Allard et al., 1994; Casademont and Miro, 2002; Neubauer, 2007; Bugger et al., 2010), and ketone body was reported to be an alternative fuel in advanced human heart failure (Aubert et al., 2016; Bedi, Snyder et al., 2016). Although the myocardial glucose uptake is augmented, glucose oxidation and its contribution to ATP production is markedly decreased (Paolisso et al., 1994; Moravec et al., 1996; Funada et al., 2009; Mori et al., 2013; Zhabyeyev et al., 2013; Zhang et al., 2013). *Where does the glucose go*? In both humans and animals, there is increased flux of glycolysis (Allard et al., 1994; Degens et al., 2006; Akki et al., 2008; Symons and Abel, 2013) and plasma concentrations of lactate during heart failure (Diakos et al., 2016; Fillmore et al., 2018). It is therefore apparent that mitochondrial oxidation shifts to glycolysis as a major metabolic reprogramming event in the failing heart.

Increased glycolysis is linked to augmented production of endogenous lactate. In addition, the intracellular lactate shuttle process is inhibited by impaired activity of mLOC, manifested by the progressive impairment of PDH (Funada et al., 2009; Zhabyeyev et al., 2013; Zhang et al., 2013; Dodd et al., 2014; Diakos et al., 2016; Fillmore et al., 2018) and low expression of MCT (Gupte et al., 2014). Taken together, these events would give rise to the accumulation of intracellular lactate in the myocardium. This however, does not allow to conclude whether high level of intracellular lactate is good or bad! On the one hand, it has been indicated that intracellular lactate overload is able to trigger the influx of Na^+^ and Ca^2+^, which can induce a decrease in the systolic function of myocardium (Fiolet and Baartscheer, 2000; Jaswal et al., 2011). On the other hand, both basic science and clinical evidence have pointed toward an important role of lactate as a key energy substrate in the failing heart (Schurr et al., 1997; Chiolero et al., 2000; Luptak et al., 2005). A recent study described heart metabolomics profiling of the uptake and release of metabolites in patients with or without heart failure. This study showed that the failing heart nearly doubled lactate consumption compared to the normal heart (Murashige et al., 2020). The mRNA expression of MCT4 (which mediates the transmembrane transport of lactate) increased 2.5–3.5 times higher of its original level during myocardial injury (Zhu et al., 2013; Gabriel-Costa et al., 2015). Promoting the lactate transport from cytoplasm to mitochondria improved energy deficiency in heart failure (Wilson et al., 1998; McClelland and Brooks, 2002; Zhu et al., 2013). Furthermore, inhibition of MCT4 and hence lactate export in a cell model of heart failure led to further accumulation of intracellular lactate and increased lactate transport for mitochondrial oxidization (Cluntun et al., 2021).

Hyperlactatemia and lactic acidosis reflect an unbalanced state of lactate production and disposal. In long-term clinical practice, lactate is regarded as a risk factor of heart failure. In patients who suffer from heart failure and have elevated blood lactate, many clinicians may empirically consider pharmacotherapeutic interventions with agents that may modulate lactate metabolism, and vasodilators or positive inotropic drugs. However, systemic lactate deprivation is disadvantageous to myocardial energy supply in pathological conditions (Levy et al., 2007). Given that lactate concentrations in capillary, arterial, and venous blood are insufficient to distinguish excess lactate production from impaired lactate clearance, it would be imprudent to treat abnormal blood lactate levels in patients with heart failure.

### Lactate Metabolism in Diabetic State

Adaptations to long-term diabetic state induce changes in cardiac energy substrate preference. Rising circulating fatty acids (Reaven et al., 1988; Young et al., 2002; Atkinson et al., 2003), high myocardial uptake of fatty acids (Avogaro et al., 1990; Sampson et al., 2003; Bonen et al., 2004; Peterson et al., 2008), and increased myocardial fatty acid β-oxidation (Belke et al., 2000; Aasum et al., 2003; Carley and Severson, 2005; How et al., 2007; Bugger and Abel, 2014; Riehle and Abel, 2016; Kenny and Abel, 2019) are important metabolic characteristics in diabetes. In addition, there are studies reporting hyperketonemia and high myocardial ketone body utilization in poorly controlled diabetes (Lommi et al., 1996, 1997; Kodde et al., 2007). The so-called “ketone body metabolic pathway” doesn’t really exist in the cell, because ketone bodies can cross the mitochondrial membrane and the cell membrane directly through the MCTs and enter the TCA cycle (Halestrap, 2013; Felmlee et al., 2020). The flow of ketone bodies into the TCA cycle is expected to inhibit mitochondrial oxidation of glucose and lactate in cardiomyocytes. Compared to studies on fatty acid and ketone body metabolism in heart, very few studies have assessed myocardial glucose and lactate utilization in the diabetic state. It is generally accepted that acceleration of fatty acid β-oxidation and insulin resistance are related to the reduction of glucose oxidation (Randle et al., 1963; Randle, 1995, 1998; Peterson et al., 2004). With regard to myocardial lactate metabolism, several studies observed the rate of lactate efflux was greater than lactate uptake under diabetic condition (Chatham et al., 2001). Given that MCT expression in cardiomyocytes is not influenced by diabetes in rat models (Chatham et al., 1999), decreased lactate uptake might be related to the increased cytosolic NADH/NAD^+^ ratio in the diabetic state (Puckett and Reddy, 1979; Ramasamy et al., 1997; Trueblood and Ramasamy, 1998; Chatham et al., 1999). Taken together, myocardial glucose and lactate metabolism are impaired on diabetic state. Impaired insulin signaling was reported to impel the deterioration of cardiac function (Abel et al., 1999; Peterson et al., 2004; Zhang et al., 2013; Byrne et al., 2016). Patients with diabetes exhibited an increased risk of heart failure, and cardiac hypertrophy is the main pathologic change of the myocardial remodeling. As discussed, substrate switch of heart failure generally appeared as a decreased fatty acid oxidation and an increased uptake of glucose and lactate (Neubauer, 2007; Casademont and Miro; 2002, Allard et al., 1994; Krishnan et al., 2009; Bugger et al., 2010). By contrast, diabetic heart failure exhibits different substrate switch—an increase in fatty acid metabolism and a decrease in glucose and lactate metabolism (Bugger and Abel, 2014; Riehle and Abel, 2016; Kenny and Abel, 2019). It is also intriguing that these divergent cardiac energy substrate preferences result in similar myocardial remodeling.

Metformin is the most widely used oral agent for the management of type 2 diabetes. In the landmark UKPDS study, the use of metformin was reported to reduce the risk of myocardial infarction by 39% (No Authors listed., 1998). Metformin is associated with a modest increase plasma lactate levels, but the risk of lactate acidosis is rare (Abbasi et al., 2000; Liu et al., 2009; Shen et al., 2012; Koren et al., 2017). Metformin does not appear to affect myocardial lactate utilization, but has been shown to reduce the intracellular lactate shuttle and increase lactate accumulation (Madiraju et al., 2014; Duca et al., 2015; Lu et al., 2017). Given that metformin is associated with increased lactate production, and tissue hypoxia is always present during heart failure. Metformin has previously been believed to increase the risk of lactic acidosis in heart failure patients. However, in recent years, metformin has been exhibited to be safe and effective in patients with heart failure in several large retrospective studies (Misbin et al., 1998; Aguilar et al., 2011). Based on these studies, metformin has been recommended for patients with diabetes mellitus and chronic heart failure (Eurich et al., 2005, 2013). Taken together, the effects of metformin on lactate metabolism do not outweigh its the benefits in diabetic cardiomyopathy. But we have to pay more attention to metformin-associated lactic acidosis (MALA), a symptom that may occur in the clinic. The exact mechanism of MALA is still unknown, but it would be necessary to elevated blood metformin concentration and secondary obstacles of lactate production and clearance (Lucis, 1983; Owen et al., 2000; Almirall et al., 2008; Frid et al., 2010; Bridges et al., 2014). As these secondary events may be unpredictable and heterogeneous, current clinical application of metformin may be too conservative. Given that fatal consequence of MALA, metformin must be used with caution, particularly in patients with circulatory dysfunction (Buse et al., 2016).

## Conclusion

In the pursuit of an understanding of myocardial metabolism, lactate was once considered as a metabolic waste, and the metamorphosis from “ugly duckling” to “white swan” was full of frustration and ordeals. Up to time now, it is apparent that lactate has multiple identities in myocardial metabolism, including energy substrate, metabolite, signal molecule, and prognostic factor. Cardiac lactate metabolism is a dynamic process that can rapidly shift to adapt to alterations in cardiac energy requirements or changing environment. Lactate is also very important as an energy substrate in acute myocardial ischemia, heart failure, and diabetic state, and abnormal myocardial lactate metabolism is closely related to diseases. Characterization of the lactate metabolic profile of myocardium in physiological and pathological conditions may help direct future pharmacological therapies to harmonize the metabolic flexibility of the heart.

## Author Contributions

SD, LQ, ZC, CC, KW, and XZ were involved in writing of the manuscript. SH, XZ, and TW involved in the conception. XZ and TW was involved in the conception, design, and the coordination of the study. SD, LQ, and ZC were involved in the picture drawing. SD, LQ, ZC, CC, KW, SH, XZ, and TW critically reviewed the manuscript and have approved the publication of this final version of the manuscript. XZ and TW are the guarantors of this work. All authors contributed to the article and approved the submitted version.

## Conflict of Interest

The authors declare that the research was conducted in the absence of any commercial or financial relationships that could be construed as a potential conflict of interest.

## Publisher’s Note

All claims expressed in this article are solely those of the authors and do not necessarily represent those of their affiliated organizations, or those of the publisher, the editors and the reviewers. Any product that may be evaluated in this article, or claim that may be made by its manufacturer, is not guaranteed or endorsed by the publisher.
