# Corrigendum: Lactate and Myocardiac Energy Metabolism

**DOI:** 10.3389/fphys.2022.947253

**Published:** 2022-06-09

**Authors:** Shuohui Dong, Linhui Qian, Zhiqiang Cheng, Chang Chen, Kexin Wang, Sanyuan Hu, Xiang Zhang, Tongzhi Wu

**Affiliations:** ^1^ Department of General Surgery, Qilu Hospital of Shandong University, Jinan, China; ^2^ Department of Colorectal and Anal Surgery, Feicheng Hospital Affiliated to Shandong First Medical University, Feicheng, China; ^3^ Department of General Surgery, The First Affiliated Hospital of Shandong First Medical University, Jinan, China; ^4^ Adelaide Medical School and Centre of Research Excellence in Translating Nutritional Science to Good Health, The University of Adelaide, Adelaide, SA, Australia; ^5^ Endocrine and Metabolic Unit, Royal Adelaide Hospital, Adelaide, SA, Australia

**Keywords:** myocardium, cardiac metabolism, energy substrate, lactate, lactate shuttle theory, myocardial ischemia, heart failure, diabetic cardiomyopathy

In the published article, there was an error in the article title. Instead of “Lactate and Myocadiac Energy Metabolism”, it should be “Lactate and Myocardiac Energy Metabolism”.

The authors apologize for this error and state that this does not change the scientific conclusions of the article in any way. The original article has been updated.

